# Advances in mass spectrometry based strategies to study receptor tyrosine kinases

**DOI:** 10.1107/S2052252516020546

**Published:** 2017-02-23

**Authors:** Simon Vyse, Howard Desmond, Paul H. Huang

**Affiliations:** a Division of Cancer Biology, The Institute of Cancer Research, London SW3 6JB, England

**Keywords:** receptor tyrosine kinase, mass spectrometry, phosphoproteomics, signal transduction, cancer

## Abstract

Receptor tyrosine kinases (RTKs) are key transmembrane environmental sensors that are capable of transmitting extracellular information into phenotypic responses, including cell proliferation, survival and metabolism. Advances in mass spectrometry (MS)-based phosphoproteomics have been instrumental in providing the foundations of much of our current understanding of RTK signalling networks and activation dynamics. Furthermore, new insights relating to the deregulation of RTKs in disease, for instance receptor co-activation and kinome reprogramming, have largely been identified using phosphoproteomic-based strategies. This review outlines the current approaches employed in phosphoproteomic workflows, including phosphopeptide enrichment and MS data-acquisition methods. Here, recent advances in the application of MS-based phosphoproteomics to bridge critical gaps in our knowledge of RTK signalling are focused on. The current limitations of the technology are discussed and emerging areas such as computational modelling, high-throughput phospho­proteomic workflows and next-generation single-cell approaches to further our understanding in new areas of RTK biology are highlighted.

## Abstract 2

This review discusses advances in mass spectrometry-based strategies to investigate receptor tyrosine kinase signalling networks and activation dynamics.

## 1. Introduction   

Since the discovery of the epidermal growth factor receptor (EGFR) over 35 years ago, 90 genes in the human genome have been identified to encode tyrosine kinases and, among these, 58 belong to the receptor tyrosine kinase (RTK) superfamily (Robinson *et al.*, 2000 ▸). Classical features of RTKs include an extracellular ligand-binding domain, a trans­membrane region and a cytoplasmic domain which possesses tyrosine kinase activity (Lemmon & Schlessinger, 2010 ▸). Ligand binding induces receptor activation, typically *via* receptor dimerization and autophosphorylation of tyrosine residues on the intracellular tail. These phosphorylated residues function as docking sites to recruit cytoplasmic signalling and adaptor proteins, providing a scaffold from which a vast array of downstream networks can be initiated (Pawson, 2004 ▸). In this manner, RTKs act as environmental sensors that are capable of converting extracellular information into complex cellular responses.

Many RTKs orchestrate key cellular processes, including cell survival, metabolism, proliferation and migration. As a result, genetic aberrations which alter RTK expression, local­ization or regulation can contribute to malignancies, and are particularly prevalent in many cancers (Lahiry *et al.*, 2010 ▸). Given the critical role of RTKs in regulating cellular function, there has been much effort in the last three decades to gain a better understanding of RTK biology.

Technical developments in mass spectrometry (MS)-based phosphoproteomics have provided powerful tools to investigate important aspects of RTK biology. With advances in phosphopeptide-enrichment strategies and metabolic and chemical labelling techniques, large-scale quantitative phosphoproteomics has unravelled the complex and dynamic nature of RTK signalling networks. In addition, MS-based strategies to map RTK–protein interactions, their substrates and their downstream pathways have led to new insights in RTK biology, including the identification of new kinase-substrate motifs and delineation of novel mechanisms of kinase-inhibitor resistance (Huang, 2012 ▸). This technology has been particularly useful in the context of cancer research, where previously poorly understood phenomena such as kinome reprogramming and RTK co-activation have now been extensively characterized using mass spectrometry (Tan *et al.*, 2017 ▸).

Despite this progress, there are inherent limitations to MS-based approaches. Owing to the complexity and low abundance of the phosphoproteome, extensive sample-preparation workflows and high-resolution mass spectrometers are required to achieve substantial phosphosite coverage. This often results in challenges such as a lack of data reproducibility (Wolf-Yadlin *et al.*, 2007 ▸), low-throughput analysis and the need for relatively large amounts of input material (Noujaim *et al.*, 2016 ▸). In addition, in order to draw biologically meaningful conclusions from phosphoproteomic data, computational strategies are necessary to effectively interrogate RTK signalling networks and integrate this information with other high-throughput profiling data such as genomics and transcriptomics (Ren *et al.*, 2011 ▸). This review outlines the recent advances in applications of MS-based phospho­proteomics in RTK biology. We briefly discuss contemporary phosphopeptide-enrichment methods and data-acquisition strategies and their applications to characterize important aspects of RTK biology. We then offer a perspective on how next-generation MS and computational strategies may overcome the current limitations of phosphoproteomics to advance our understanding of outstanding questions in RTK research.

## 2. Mass spectrometry-based phosphoproteomics   

**Figure 1:**
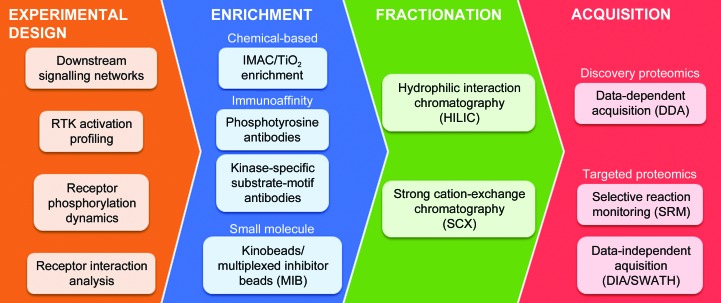
Overview of mass spectrometry-based phosphoproteomic workflows. Depending on the experimental design, there are a number of different strategies which can be chosen to enrich specific compartments of the phosphoproteome. This is commonly followed by fractionation to reduce complexity and increase coverage of complex cell lysates. Finally, the method of data acquisition will be influenced by the specific biological questions of interest when interrogating proteomic data.

Protein phosphorylation and its regulation through the recip­rocal actions of protein kinases and phosphatases play a central role in many vital cellular processes. The phosphoproteome is defined as the phosphorylated component of the proteome in tissues or cells. The development of successful phosphoproteomic methods is the result of technological and sample-preparative improvements in three areas: (i) advances in mass-spectrometer technology leading to high mass accuracy and rapid scan speeds, (ii) the development of selective enrichment methods for phosphopeptides and (iii) improved methods of phosphopeptide data acquisition and quantitation. The central challenge in phosphoproteomics is the low abundance of phosphorylated species within the proteome. A minority of proteins are phosphorylated in the cell at any given time and those proteins that are phosphorylated are often present at low stoichiometry, frequently as low as 1% (Wu *et al.*, 2011 ▸). Thus, without some form of prior enrichment and the use of highly sensitive mass spectrometers, few phosphorylated species would reliably be detected in direct liquid chromatography–mass spectrometry (LC-MS) experiments. This section describes the most commonly used phosphopeptide-enrichment methodologies as well as current data-acquisition strategies (Fig. 1 ▸).

### 2.1. Enrichment methodologies   

**Figure 2:**
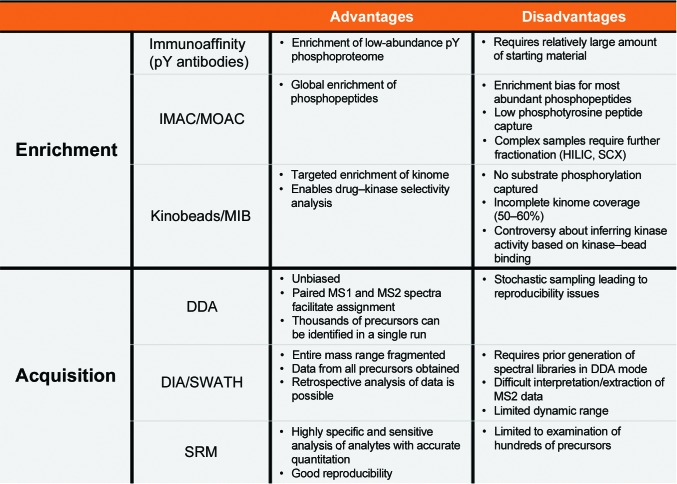
Advantages and disadvantages of enrichment and acquisition methods in phosphoproteomic workflows. A comparison of the benefits and drawbacks of chemical, immunoaffinity and small-molecule-based phosphoproteome enrichment and data-dependent (DDA), data-independent (DIA) and selective reactive monitoring (SRM) acquisition methods, which must be considered when designing phosphoproteomic experiments.

Methods for phosphoprotein enrichment are broadly split into chemical-based chromatography approaches, immune affinity-purification methods and small-molecule binding strategies (Fig. 2 ▸).

A number of chemical-based chromatography approaches have been developed to enrich phosphoserine- and phosphothreonine-containing peptides while reducing nonspecific binding, particularly by the more abundant acidic peptides. Immobilized metal-affinity chromatography (IMAC) is an established method in phosphoproteomics which uses metal ions such as Fe^3+^, Zr^4+^ or Ga^3+^ that have been chelated to suitable resins to selectively retain negatively charged phospho­peptides (Ficarro *et al.*, 2002 ▸). However, it should be noted that such resins also bind tightly to acidic peptides, and methods have been described to reduce this nonspecific binding: for example, reducing the sample pH so that the phosphate group but not glutamate or aspartate side chains remain negatively charged or the methyl­esterification of the side chains of acidic residues to reduce the binding of these acidic peptides to the IMAC resin (Ficarro *et al.*, 2002 ▸). Metal oxide affinity chromatography (MOAC) using titanium dioxide (TiO_2_) has also been extensively used for the affinity isolation of phosphopeptides. The method makes use of the affinity of oxygen in phosphoryl groups for the metal (Sano & Nakamura, 2004 ▸). It has been shown that IMAC and TiO_2_ enrich different spectrums of phosphopeptides, and a sequential combination of these two approaches has been employed to gain an increase in the coverage of the phosphoproteome (Engholm-Keller *et al.*, 2012 ▸).

Because of the large number of different phosphopeptides present in cell and tissue lysates, additional fractionation is often carried out to increase the depth of phosphoproteome analysis. Methods orthogonal to conventional reverse-phase chromatography are widely used. These include strong cation-exchange chromatography (SCX) and hydrophilic interaction chromatography (HILIC). SCX methods rely on the relatively poor retention of negatively charged tryptic phosphopeptides by the resin at low pH compared with the bulk of unmodified tryptic peptides. The method was first applied to the large-scale characterization of the HeLa cell nuclear phospho­proteome to identify >2000 phosphosites on 967 proteins (Beausoleil *et al.*, 2004 ▸). HILIC is based on normal-phase chromatography (except that it is compatible with mobile phases containing water) using any of a variety of polar bonded phases in which solutes are eluted in order of increasing hydrophilicity. HILIC exploits the strong hydrophilicity of phosphopeptides, and typically phosphopeptides elute in the middle of the chromatogram, allowing substantial fractionation and subsequent enrichment using either IMAC or TiO_2_ (McNulty & Annan, 2008 ▸).

Immunoaffinity-based approaches are primarily employed for the enrichment of tyrosine-phosphorylated peptides using a number of commercially available pan-specific phospho­tyrosine antibodies. Unlike phosphoserine and phosphothreonine modifications, which represent 90 and 10% of the phosphoproteome, respectively, tyrosine phosphorylation is a relatively small fraction, accounting for less than 0.1% of protein-phosphorylation events in the cell (Hunter & Sefton, 1980 ▸). Immunoprecipitation using phosphotyrosine antibodies has been used for the enrichment of both tyrosine-phosphorylated proteins and peptides from cell lysate and tissues (Rush *et al.*, 2005 ▸). The typical workflow for analysis of tyrosine phosphorylation is as follows. The cell lysate is extracted prior to proteolysis and the resulting digest is then subjected to immunoprecipitation with phosphotyrosine antibodies. Further polishing of the immunoprecipitated fraction can be carried out using IMAC or TiO_2_ to remove any non­specific peptide binding. This method has since been applied to examine dynamic phosphotyrosine signalling in many RTK pathways (such as ErbB, c-MET, PDGFR and FGFR) as well as the analysis of cell lines and tissues. In the first large-scale analysis of phosphotyrosine signalling in a panel of cell lines and tissues, Rikova and coworkers analysed 41 non-small lung cell carcinoma cell lines (NSCLCs) and over 150 tumours using this approach, and identified a total of 4500 tyrosine-phosphorylation sites on more than 2700 proteins (Rikova *et al.*, 2007 ▸). This study provided the first demonstration that multiple RTKs are activated simultaneously in cancer cell lines and tissues, and provided the foundation for future studies on the mechanisms of RTK co-activation in cancer (Huang *et al.*, 2010 ▸). The combined use of chemical-based enrichment and phosphotyrosine immunoprecipitation has been shown to achieve an extremely high depth of phosphoproteome coverage. For instance, using a combination of SCX, TiO_2_ and phospho­tyrosine antibody enrichment, Sharma and coworkers dentified more than 38 000 phosphosites from 51 000 peptides in HeLa cells over 17 d of MS acquisition time (Sharma *et al.*, 2014 ▸).

While phosphorylations on serine and threonine residues are the predominant modifications present in the cellular phosphoproteome, selective antibodies to phosphoserine or phosphothreonine residues are not available owing to the small size of the modified residue and consequent lower antigenicity. However, a number of high-quality antibodies have been raised against extended serine- and threonine-phosphorylation motifs. Examples include the substrate motifs for cyclin-dependent kinases (pS/pTP), the AKT kinase motif (R*x*R*xx*pS/pT), and ATM and ATR (pS/pTQ), among others (Joughin *et al.*, 2009 ▸; Zhang *et al.*, 2002 ▸; Matsuoka *et al.*, 2007 ▸). As with pan-specific phosphotyrosine antibodies, these kinase substrate motif antibodies are used in immunoprecipitation strategies to enrich phosphopeptides containing the motif of interest. For example, ATM/ATR substrate antibodies have been deployed to characterize the cellular response to DNA damage in 293T cells subjected to ionizing radiation, which led to the identification of more than 900 regulated phosphorylation sites on >700 proteins (Matsuoka *et al.*, 2007 ▸). Many of these newly identified proteins are of unknown functions and have subsequently been shown to be important for the DNA damage-repair response (Huang & White, 2008 ▸; Matsuoka *et al.*, 2007 ▸).

Since many of the biologically significant phosphorylation events occur in kinases, a number of affinity methods have been developed to enable the enrichment and characterization of the kinome in cells and tissues. By subjecting lysates to immobilized panels of promiscuously binding kinase inhibitors, these methods exploit the conserved nature of the ATP-binding site present in all kinases to pull down a large fraction of the kinases found in the cell. While these assays are not strictly phosphopeptide-enrichment methods, they provide complementary information about the protein abundance and potentially activation states of different kinases in cells and tissues. Bantscheff and coworkers used this approach to pull down a large fraction of the total kinome with a panel of seven immobilized kinase inhibitors chosen on the basis of their broad and complementary specificity (Bantscheff *et al.*, 2007 ▸). Using these inhibitors (termed kinobeads) in pull-down experiments, they identified 174 kinases from HeLa cell extracts and a similar number from K562 cells. From a panel of 14 cell lines and tissues they were able to characterize a large fraction (307 of 518) of the kinome. There is some controversy as to whether these binding events represent the active complement of the kinome. The assumption that such resins predominantly bind kinases in their active form was challenged when Ruprecht and coworkers showed in a systematic study that the immobilized kinase inhibitors showed no preference for kinase activation (Ruprecht *et al.*, 2015 ▸).

A more recent variant of the kinobead procedure uses a combination of inhibitors with broad kinase selectivity as well as clinically available tyrosine kinase inhibitors (TKIs). This technique is known as multiplexed kinase inhibitor beads and mass spectrometry (MIB/MS), and has been used to characterize the reprogramming of the receptor tyrosine kinome in response to targeted therapy in breast cancer (Stuhlmiller *et al.*, 2015 ▸; Duncan *et al.*, 2012 ▸). Duncan and coworkers employed this approach to demonstrate that the RTK profile significantly alters in response to MEK inhibitor treatment in the SUM159 triple-negative breast cancer cell line (Duncan *et al.*, 2012 ▸). They termed this phenomenon kinome reprogramming. This reprogramming induced resistance to MEK inhibition, and the use of RTK inhibitors (sorafenib and foretinib) to overcome kinome reprogramming restored sensitivity to the MEK inhibitor AZD6244. These RTK reprogramming effects appear to be a general cellular response to targeted therapy, as subsequent studies have shown that similar reprogramming events are responsible for kinase-inhibitor resistance in ErbB2-positive breast cancer cell lines and BET bromodomain-inhibitor resistance in ovarian carcinoma cell lines (Stuhlmiller *et al.*, 2015 ▸; Kurimchak *et al.*, 2016 ▸). These studies highlight the power of affinity pull-down experiments in identifying novel RTK-based strategies to overcome targeted therapy resistance in multiple cancer types.

Finally, a chemical genetic approach which employs kinases engineered with the ability to use analogues of adenosine 5′-triphosphate (ATP), so-called analogue-sensitive (AS) kinases, has been employed to isolate kinase-specific substrates for downstream phosphoproteomic analysis. An analogue-sensitive ERK2 (AS-ERK2) was used by Carlson and White to identify ERK substrates by tagging them with γ-thiol-phosphate ATP analogues in NIH 3T3-L1 fibroblasts (Carlson & White, 2012 ▸). Following the capture of thiophos­phorylated substrate residues, coupled with IMAC to reduce nonphosphorylated background peptides, Carlson and White were able to establish 98 sites on 80 proteins phosphorylated by AS-ERK2, including a novel and functionally relevant phosphorylation of the E26 (ETS) domain-containing protein ETV3. This study exemplifies the use of AS kinases for focused kinase-substrate studies; however, caution must be taken to validate these findings in order to rule out the potential false positives which might arise by the nonspecific utilization of ATP analogues by endogenous kinases within the cell.

### 2.2. Data-acquisition methodologies   

**Figure 3:**
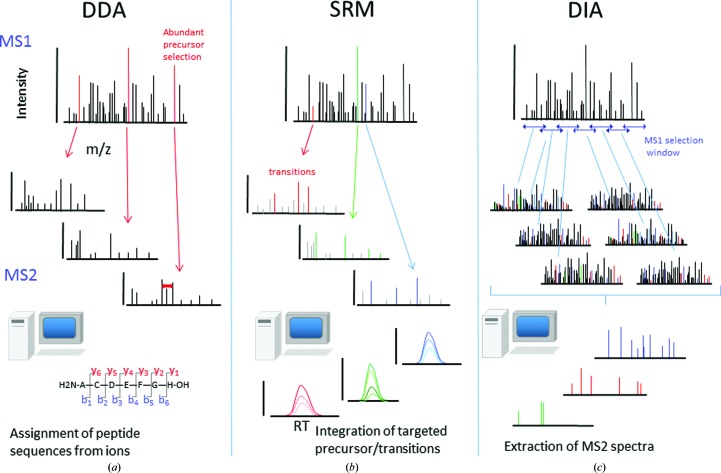
Schematic comparison of mass-spectrometric data-acquisition methodologies. (*a*) DDA: precursors identified in the first MS1 stage are selected for MS2 fragmentation on the basis of abundance. Software matches the masses to the database (*in silico* ‘trypsinized’ proteins). This is the standard discovery mode allowing the identification of novel proteins and phosphorylation sites. (*b*) SRM: precursors chosen on basis of prior discovery experiments in the MS1 stage; following fragmentation, signature MS2 peaks are also selected. The integration of these transitions can be used for quantitation. (*c*) DIA: no precursor selection in the MS1 stage; instead, all ions in wide overlapping mass windows (typically 25 mass units) over the whole mass range (from 400 to 1200 *m*/*z*) are fragmented. Using spectral libraries obtained in DDA experiments, MS2 spectra corresponding to specific peptides can be extracted.

The data-acquisition methodology used to acquire phosphoproteomic data is a critical consideration that influences the type of data generated in MS experiments, and the choice of which method to use is largely dependent on the nature of the proteomic application of interest (Fig. 2 ▸). For many years, MS-based proteomics have been carried out using data-dependent acquisition (DDA; Fig. 3 ▸ *a*). In this approach, peptides eluting from the liquid-chromatography (LC) column become ionized in the mass spectrometer (Aebersold & Mann, 2016 ▸). The mass/charge (*m*/*z*) ratio is determined in the first stage of the instrument (the MS1 stage) and typically the 10–20 most abundant precursors are selected for fragmentation in the second MS2 stage. Conventional LC conditions mean that the tens of thousands of peptides present in a complex sample will elute over the course of a 1–4 h gradient. In this approach, the selection of peptide precursor ions tends to be a stochastic process (albeit biased to the more abundant peptide species) such that the overlap between two technical replicates (in peptides identified) is routinely less than 70% (Wolf-Yadlin *et al.*, 2007 ▸). This stochastic process means that many peptides in complex mixtures will go undetected and peptides will not be reproducibly detected. The main advantage of this method is that it is unbiased, facilitating the discovery of new phosphorylation events in a single LC-MS run. As mass-spectrometer technology improves and scan speeds and cycle times become shorter and detectors more sensitive, it is anticipated that some of these limitations of reproducibility will gradually diminish.

A second strategy for data acquisition is targeted proteomics based on selective reaction monitoring (SRM; Fig. 3 ▸ *b*); which utilizes the knowledge gained from DDA experiments to generate *in silico* peptide libraries that enable the specific targeting and quantification of several hundred phosphoryl­ated peptides simultaneously in a single LC-MS experiment. These SRM experiments are typically carried out on a triple-quadrupole mass spectrometer and specific precursor ions (corresponding to peptide precursors of interest previously identified in DDA discovery experiments) are selected in the first quadrupole. These selected precursors pass into the second quadrupole, where they are fragmented and all precursors outside of the narrow mass-selection window are discarded. In the final stage of the mass spectrometer, selected fragments of interest are isolated and measured in the final quadrupole (Carr *et al.*, 2014 ▸). Because this strategy employs an *a priori*-defined *in silico* library of peptides, the lack of reproducibility associated with stochastic sampling in DDA is avoided, leading to a close to 90% overlap between peptides identified in technical replicates. One of the early applications of this strategy to RTK signalling was performed by Wolf-Yadlin and coworkers, who utilized SRM to quantify tyrosine signalling downstream of EGF stimulation in human mammary epithelial cells (Wolf-Yadlin *et al.*, 2007 ▸). Here, the authors ‘tracked’ 222 tyrosine-phosphorylation sites and showed that while typical DDA strategies led to poor reproducibility of 34% across four replicates, SRM was superior in its ability to reproducibly quantify 88% of all the phosphoryl­ation sites monitored. While SRM generates highly reproducible data sets, unlike DDA-based approaches, the development of high-quality assays requires significant optimization and lead time (Carr *et al.*, 2014 ▸). Furthermore, these assays have a limited depth of phosphoproteome coverage, often restricted to several hundred phosphorylation sites (Kennedy *et al.*, 2016 ▸). Finally, owing to their reliance on *a priori in silico* libraries, SRM approaches do not allow the discovery of new proteins and post-translational modifications that are commonly associated with DDA.

An alternative strategy to DDA and SRM is data-independent acquisition (DIA), which is also known as sequential window acquisition of all theoretical fragment-ion spectra (SWATH; Fig. 3 ▸ *c*). In this approach, all peptide precursor ions present in wide overlapping (typically 20 Da) windows across the whole mass range are fragmented (Hu *et al.*, 2016 ▸), generating all possible precursor fragment-ion (MS/MS) spectra. The major challenge with DIA is the requirement to extract the information for a given precursor from the resulting complex MS2 data, which will contain thousands of fragment ions. As a result, this data-acquisition methodology relies heavily on bioinformatics tools to deconvolute complex mass spectra; for instance, using data from prior experiments in DDA mode to generate spectral libraries which can be used in the interrogation of DIA data (Röst *et al.*, 2014 ▸). The main advantage of this method is that, unlike DDA approaches, the DIA data can be retrospectively interrogated for proteins of interest. Employing DIA, Parker and coworkers demonstrated the utility of this approach to quantify the phosphoryl­ation of multiple components of the insulin receptor signalling cascade and were able to demonstrate that AKT2-dependent phosphorylation of GAB2 inhibited EGF signalling and promoted insulin signalling in a 14-3-3 binding manner (Parker *et al.*, 2015 ▸). DIA methodology is still very much in its infancy and it is anticipated that as the technology and software become more widespread in use, this approach will be deployed for more RTK studies in the near future

## 3. Mass spectrometry unveils new RTK biology   

**Figure 4:**
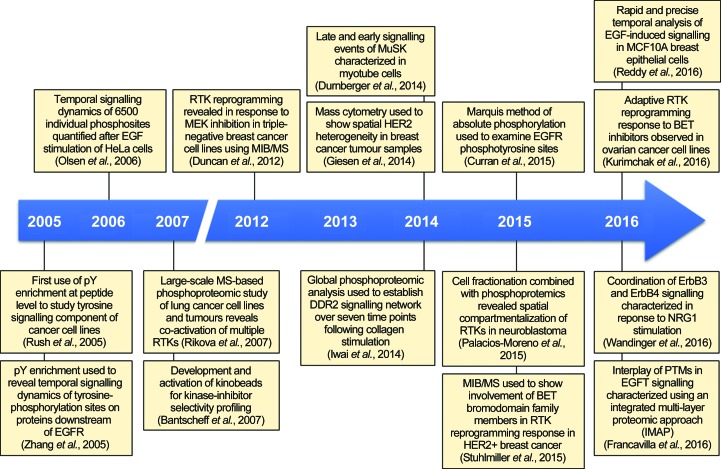
Advances in understanding RTK biology using mass spectrometry-based phosphoproteomic studies. A timeline of key studies which illustrate the development of MS-based phosphoproteomics and their application in advancing our knowledge of RTK biology. The timeline depicts the pioneering phosphoproteomic studies performed a decade ago in addition to highlighting novel and innovative research from the last five years.

In the last decade, the use of MS-based phosphoproteomics has been integral to RTK research (Fig. 4 ▸). This includes the characterization of the mechanisms of action of many RTKs that are deregulated in cancer and other diseases such as diabetes, studies of resistance mechanisms to kinase inhibitor therapy and modelling the kinetics of RTK signalling (Huang, 2012 ▸; Tan *et al.*, 2017 ▸). In this section, we highlight the most recent applications of MS-based phospho­proteomics to unravel new aspects of RTK biology.

### 3.1. New insights in EGFR phosphorylation dynamics and downstream signalling   

EGFR is the prototypical member of the RTK superfamily and has been the subject of multiple phosphoproteomic studies over the past decade (Mertins *et al.*, 2012 ▸; Olsen *et al.*, 2006 ▸; Zhang *et al.*, 2005 ▸). By quantifying the dynamics associated with EGFR activation and its downstream signalling in a range of cell lines, these studies have detailed the canonical EGFR signalling networks, which has led to important applications in human health and disease. Despite the wealth of prior knowledge associated with this well studied receptor, there are several outstanding fundamental questions that remain unanswered. For instance, all studies to date have focused on signalling that occurs on the minutes to hours timescale after ligand stimulation, and the nature of the immediate early signalling networks remains unknown. Furthermore, it has been a challenge to directly compare the phosphorylation levels across different sites on the EGFR protein since all published studies have thus far relied on relative quantification to a reference sample/condition. Given that site-specific receptor phosphorylation has a direct impact on adaptor recruitment and propagation of downstream signalling, the ability to map absolute levels of receptor phosphorylation has profound implications for our understanding of EGFR signalling.

Recent studies have started to shed light on these important questions. For instance, Reddy and coworkers focused their efforts on mapping the phosphotyrosine signalling events at high temporal resolution within a timeframe of seconds (Reddy *et al.*, 2016 ▸). Using the MCF10A cell line as a model system, they employed phosphoproteomics to characterize the signalling profiles for the first 80 s after EGF stimulation at 10 s intervals over eight different growth-factor concentrations. They quantified several hundred tyrosine-phosphoryl­ation sites and were able to show that EGFR was capable of initiating downstream signalling almost immediately after ligand activation. They also identified very early phosphorylation changes on proteins not previously known to be in the EGFR pathway, such as the cytoskeletal components cortactin, plakophilin and tensin. By integrating these phosphorylation data with localization measurements using proximity ligation assays, the authors demonstrated that the binding of the receptor to adaptor proteins such as SHC and GAB1 occurs on similar timescales as phosphorylation, suggesting that adaptor recruitment to the receptor may be the primary rate-limiting step in regulating early phospho­rylation events.

To tackle the issue of absolute phosphorylation measurements in EGFR, Curran and coworkers developed a method which combined chemical labelling with isotopically labelled synthetic peptides to generate internal standard curves for phosphopeptides in the EGFR signalling network (Curran *et al.*, 2015 ▸). They called this approach the multiplex method for absolute quantitation of peptides and post-translational modifications (MARQUIS). By analysing MCF10 cells stimulated with EGF, the authors demonstrated for the first time that the Tyr1148 site was phosphorylated at a fivefold higher level than Tyr1173, Tyr1068 or Tyr1045. This was an interesting finding, as Tyr1173 rather than Tyr1148 is classically considered to be the dominant autophosphorylation site on EGFR (Voldborg *et al.*, 1997 ▸). By comparing the signalling dynamics across three distinct EGF family ligands (EGF, TGFα and amphiregulin), the authors determined that the comparative phosphorylation pattern of different receptor sites remained quantitatively consistent regardless of the ligand used. This finding suggests that the biological responses associated with different EGF ligands may be the result of quantitative differences in receptor phosphorylation (changes in absolute levels of phosphorylation at different sites) rather than qualitative differences (the activation of different phosphorylation sites on the receptor by different ligands). By utilizing these new approaches to analyse EGFR signalling, these two studies have challenged the established dogma in the field, generating new insights into EGFR biology for further investigation.

### 3.2. Defining the canonical signalling in poorly characterized RTKs   

Out of the 58 RTKs in the human genome, there is still a large fraction of receptors that remain poorly characterized and for which the canonical signalling pathways are unknown (Lemmon & Schlessinger, 2010 ▸). Several recent studies have highlighted the utility of phosphoproteomics to map, for the first time, the downstream signalling pathways in a subset of these poorly characterized receptors, including DDR2, MuSK and ErbB4 (Iwai *et al.*, 2013 ▸; Durnberger *et al.*, 2014 ▸; Wandinger *et al.*, 2016 ▸).

The discoidin domain receptors (DDRs) are a class of RTKs, comprising DDR1 and DDR2, that are activated by binding to collagen rather than growth-factor ligands (Iwai *et al.*, 2014 ▸). Upon ligand stimulation, these receptors display a unique delayed activation profile that results in the initiation of phosphorylation only hours after ligand engagement. Furthermore, unlike other RTKs, DDR signalling is sustained over several days with no apparent negative regulatory mechanisms. The receptor-phosphorylation sites and signalling pathways activated by the DDRs are poorly annotated, making it challenging to define the mechanistic basis of the cellular phenotypes associated with this class of receptors. Combining IMAC enrichment and phosphotyrosine immunoprecipitation, our laboratory has performed a global phosphoproteomic analysis to characterize the signalling networks activated by DDR2 over seven time points (0–24 h) after collagen stimulation (Iwai *et al.*, 2013 ▸). Using multiple clustering strategies, a subset of tyrosine-phosphorylated proteins was identified as candidate substrates of DDR2 activation, including the SHP2 protein tyrosine phosphatase. Having determined the key receptor-phosphorylation sites and signalling pathways activated by DDR2, we further demonstrated that the mechanism of action for kinase-domain mutations of DDR2 in lung cancer was kinase inactivation and the inability to initiate receptor phosphorylation and downstream signalling. This resulted in a loss of receptor function, alleviating the growth-suppressive properties of DDR2, and an increase in colony formation by these cancer-associated mutants (Payne & Huang, 2014 ▸).

The muscle-specific kinase (MuSK) is required for the formation of the neuromuscular synapse (Kim *et al.*, 2008 ▸). It is activated by the heparin sulfate proteoglycan agrin in complex with the Lrp4 receptor (Kim *et al.*, 2008 ▸). Durnberger and coworkers used myotube cells as a model system to study the global phosphorylation effects of MuSK activation by agrin over four time points (0–240 min; Durnberger *et al.*, 2014 ▸). Employing a combination of TiO_2_ enrichment and SCX fractionation, the authors quantified >10 000 phosphopeptides, of which 203 phosphopeptides on 152 proteins were significantly regulated by agrin stimulation of MuSK. By subjecting these phosphopeptides to *K*-means clustering, they identified both early-response (15 min) and late-response (1 and 4 h) clusters. Pathway-enrichment analysis of early signalling events identified a strong enrichment of actin cytoskeletal proteins, which included paxillin, talin and vinculin, among others. These proteins are known to play a key role in the regulation of the cytoskeleton, which is consistent with the knowledge that neuromuscular synapse formation is accompanied by significant cytoskeletal reorganization. This study represents the first analysis of MuSK RTK signalling and presents a useful paradigm for further functional analysis of the relationship between downstream effector signalling and neuromuscular synapse cytoskeletal reorganization.

The EGFR or ErbB receptor class is composed of four family members: EGFR, ErbB2, ErbB3 and ErbB4 (Citri & Yarden, 2006 ▸). ErbB4 remains the most poorly characterized member of this class of receptors. ErbB4 is capable of forming homodimers and heterodimers, and it has been shown that the neuregulin-1 (NRG1) ligand promotes the homodimerization of ErbB4 as well as the heterodimerization of ErbB4 with ErbB3 (Citri & Yarden, 2006 ▸). However, the downstream signalling events activated by ErbB4 homodimers and ErbB3/ErbB4 heterodimers are unknown. In a recent study, Wandinger and coworkers ectopically expressed ErbB4 alone or ErbB3 and ErbB4 in the murine Ba/F3 model cell-line system and analysed the phosphorylation signals upon NRG1 stimulation (Wandinger *et al.*, 2016 ▸). Interestingly, the co-expression of ErbB3 in combination with ErbB4 promoted enhanced proliferation compared with cells expressing ErbB4 alone, suggesting that the heterodimer induces more potent cellular signalling. Using MS-based phosphoproteomics, the authors identified >9600 phosphosites, of which 492 were significantly altered in ErbB3/ErbB4-expressing Ba/F3 cells. Kinase substrate-motif analysis of these phosphosites found that the AKT substrate motif was enriched in response to NRG1 stimulation. Comparing the ErbB3/ErbB4 and ErbB4-specific phosphorylation data identified 54 phosphosites that were distinct between the two cell lines. Importantly, all of these sites were found to be more highly phosphorylated upon the co-expression of ErbB3, suggesting that ErbB3 serves an important function as a broad amplifier of ErbB4 signalling.

Taken together, these three studies highlight the utility of phosphoproteomics to elucidate the signalling networks of poorly characterized RTKs. There remain a large number of RTKs for which the canonical signalling networks are unknown, including INSRR, MER, LTK and the majority of the Eph receptors, among others, and further efforts to curate the downstream phosphorylation events activated by these receptors will be necessary.

### 3.3. Interplay of EGFR post-translational modification events   

Phosphorylation is one of many post-translational modifications (PTMs) that are important for signal transduction, and thus phosphoproteomics data only present one facet of complex cellular signalling systems. In addition to phosphoryl­ation, other prominent protein-modification events include glycosylation, ubiquitination and acetylation. Several MS-based approaches have been developed to integrate phosphorylation analysis with other PTMs. Mertins and coworkers proposed a strategy of serial enrichments of different post-translational modifications (SEPTM), which uses MS to simultaneously study protein phosphorylation, ubiquitination and acetylation. The technique requires a large amount of starting material and rigorous sample fractionation and serial enrichment, using IMAC to enrich for phosphopeptides and antibodies to pull down ubiquitinated and acetylated peptides, leading to a deep interrogation of multiple PTMs from a single biological sample (Mertins *et al.*, 2013 ▸). Using this approach, they identified 20 000 phosphosites, 15 000 ubiquitination sites and 3000 acetyl­ation sites in a single experiment. By correlating changes in protein abundance and PTMs in Jurkat cells treated with bortezomib, a proteasome inhibitor, the authors were able to isolate six functional major nodes which were co-regulated and associated with key cellular process including cell cycle, transcription and proteasomal regulation.

More recently, Francavilla and coworkers expanded on this strategy and applied it in the study of EGFR signalling (Francavilla *et al.*, 2016 ▸). The authors utilized mass spectrometry to analyse the ubiquitinome, phosphoproteome, interactome and proteome alterations in HeLa cells stimulated with the EGFR ligands EGF and TGFα. They termed this strategy an integrated multi-layered proteomics approach (IMPA). EGF and TGFα elicit distinct biological phenotypes, signalling and receptor trafficking responses downstream of EGFR activation (Roepstorff *et al.*, 2009 ▸). For instance, EGF promotes EGFR degradation after internalization, while TGFα induces the recycling of active receptor. The authors subjected cells to stimulation with either of the two ligands for 1, 8, 40, 90 min or 72 h and performed multi-stage enrichment of ubiquitinated peptides by immunoprecipitation and phosphorylation by immunoprecipitation and TiO_2_ enrichment. They identified >5500 phosphosites on 1949 proteins, of which 23% were regulated by ligand addition, and 1311 ubiquitin­ated peptides on 782 proteins, of which 17% were ligand-regulated. Furthermore, they conducted receptor-interaction analysis by co-immunoprecipitation of endogenous EGFR and also quantified alterations in the proteome at 72 h after stimulation. Using these disparate data sets, they focused on 67 proteins that were regulated in more than one data set. They showed that EGF and TGFα differed in their temporal regulation of global ubiquitination to a greater extent than global phosphorylation levels, with TGFα promoting enhanced cellular ubiquitination. Furthermore, there appears to be crosstalk in the regulation of both the cellular ubiquitination and phosphorylation machinery, where the authors demonstrated that ubiquitin E3 ligases and deubiquitinating enzymes are phosphorylated, while kinases and phosphatases are ubiquitinated. The presence of multiple PTM data sets also facilitates the assessment of PTM crosstalk on the 260 proteins that were both phosphorylated and ubiquitinated. Of these proteins, only 25 were regulated at the level of both PTMs, with EGF stimulating phosphorylation at early time points (within 8 min) and TGFα activating both sustained phosphorylation and prolonged ubiquitination; highlighting the opposite PTM profiles in response to the two EGFR ligands. These findings shed light on the complex interplay and crosstalk between different PTMs in signalling networks and suggest that future experiments will need to consider the effects of both phosphorylation and ubiquitination in the regulation of RTK signalling. On a more general note, while increasing the number of PTM ‘layers’ analysed by MS will present challenges in big-data analysis and interpretation, understanding the roles of multiple PTMs and their crosstalk in signalling networks will be necessary in order to gain a more holistic understanding of biological processes regulated by RTKs.

## 4. Future perspectives   

Our current understanding of the signalling dynamics in many RTKs has relied on the ability to perform quantitative, large-scale global phosphorylation analysis. However, there are still a number of important questions which remain unanswered. Emerging technologies which improve upon current MS-based approaches will provide new opportunities to investigate burgeoning areas of RTK research.

### 4.1. Computational modelling of phosphoproteomic data   

There is a need to develop better computational methods to interrogate phosphoproteomic data. A major challenge in dealing with large phosphoproteomic data sets is finding an effective way to distil key interactions to better understand RTK signalling dynamics. Several research groups have begun to apply innovative computational strategies to model RTK networks and integrate phosphoproteomics with other large data sets. Terfve and coworkers developed a method named phosphorylation networks for mass spectrometry (PHONEMeS) to investigate drug perturbations of cell signalling in the context of known interactions (Terfve *et al.*, 2015 ▸). The authors constructed signalling networks using logic model building and training to integrate known or predicted kinase–phosphatase and kinase–substrate interactions from multiple data sources with newly acquired phosphoproteomic data. This type of analysis is capable of framing phosphoproteomic data onto a network of known protein relationships, but emphasizes biologically meaningful signalling routes instead of focusing on key interaction nodes. Analysing phosphoproteomic data using this approach can provide new insights that might otherwise be missed by using the analysis of protein–protein interaction databases such as STRING (Szklarczyk *et al.*, 2015 ▸).

Another group has developed a computational method designed to deal with a common problem in MS-based phosphoproteomic data sets, which is handling false-negative results (Grimes *et al.*, 2013 ▸). Missing values in data sets often result as a byproduct of sample loss during preparation and enrichment as well as stochastic sampling in the DDA mode. Instead of imputing missing values, Grimes and coworkers calculated statistical relationships between the observed values using protein–protein interaction data and pattern recognition to create protein network clusters. In a follow-up study, this technique was applied to phosphoproteomic data in neuroblastoma cell lines, where endosomal and detergent-resistant membrane cell fractions were isolated (Palacios-Moreno *et al.*, 2015 ▸). By examining interactions between co-clustering groups of proteins, the authors were able to elucidate an intricate crosstalk pattern between distinct groups of tyrosine kinases. In addition, this study addressed a poorly understood area of RTK biology, the regulation of RTK signalling networks *via* intracellular spatial compartmentalization. Perturbation of neuroblastoma cells with TKIs or stimulation with growth factors revealed distinct enrichment of specific RTKs to different intracellular compartments. Although it is still too early to draw general conclusions about how RTK spatial distribution regulates signalling networks, it is likely that future in-depth studies using these new computational approaches will generate new insights that will resolve the regulation of RTK signalling dynamics at this level of spatial detail.

### 4.2. Increasing the throughput of phosphoproteomics experiments   

In the context of cancer biology, there is an urgent need to develop rapid and comprehensive profiling of RTK signalling in large-scale panels of cell lines and tumours. Unlike other contemporary profiling approaches such as DNA and RNA sequencing, proteomics lacks the throughput required to generate the high-density phosphoprofiles that are necessary for understanding and targeting disease. Identifying RTK signalling profiles on a patient-by-patient basis is required to contribute to ongoing efforts to develop personalized medicine in the form of tailored treatment strategies. To address this challenge, Humphrey and coworkers recently developed a high-throughput, scalable platform for phosphoproteomics termed EasyPhos (Humphrey *et al.*, 2015 ▸). This workflow was optimized to allow proteome digestion and phosphopeptide enrichment in a 96-well format, facilitating the processing of multiple samples in parallel. Using this method, the authors were able to reach a depth of >10 000 phosphosites in mouse liver, kidney and brain tissue samples over the course of 1.5 d of mass-spectrometry acquisition. Such high-throughput phosphoproteomics techniques will speed up the translation of routine and affordable tumour phosphoprofiling in a clinical setting (Noujaim *et al.*, 2016 ▸). In addition, the study demonstrates the suitability of this platform for performing large-scale RTK signalling experiments. The authors were able to quantify signalling events downstream of the insulin receptor in mouse liver tissue across 11 stimulation time points with 6–10 biological replicates per time point. The ability to perform phosphoproteomics on this scale will facilitate experiments designed to robustly interrogate temporal RTK signalling dynamics with greater detail and precision.

### 4.3. Resolving single-cell RTK signalling and population heterogeneity   

One drawback of current phosphoproteomics strategies is that these measurements routinely require large amounts of starting material, and as a result are often carried out with high cell numbers or entire tissue sections. This limits the phosphorylation information generated to population-level measurements lacking resolution at the single-cell level. However, it is now evident that cell lines and tumours display significant heterogeneity in RTK expression and activation and are composed of several distinct subpopulations: a phenomenon first documented in glioblastoma (Snuderl *et al.*, 2011 ▸) and more recently described in colorectal tumour specimens by the Clinical Proteomic Tumour Analysis Consortium (CPTAC; Gajadhar *et al.*, 2015 ▸).

Recent advances in mass cytometry increases the capability to collect measurements from tissue subpopulations with fine detail, even down to the single-cell level. Mass cytometry derives from the principles of flow cytometry, but instead of using fluorophore-tagged antibodies for staining, antibodies are conjugated with metal-isotope tags (Bendall *et al.*, 2011 ▸). Detection of these mass-tagged antibodies is achieved through cytometry by time of flight (CyTOF). TOF mass spectrometers are able to distinguish ‘nonbiological’ rare-earth metal isotopes, creating the potential to carry out multiplexed measurement of up to 100 analytes from a single sample: a number which is expected to increase as the technology develops (Angelo *et al.*, 2014 ▸). CyTOF can be combined with high-resolution laser ablation of tissue samples prepared in a similar manner to immunohistochemistry (IHC) staining. Using this approach, Giesen and coworkers were able to detect 32 proteins and phosphorylation sites simultaneously on breast cancer tissue, with a resolution of 1 µm (Giesen *et al.*, 2014 ▸). A similar approach called multiplexed ion-beam imaging (MIBI) has been used to achieve an even greater resolution of 50 nm (Angelo *et al.*, 2014 ▸). The images produced look similar to traditional IHC staining and use software which overlays quantitative mass-spectrometry data onto their spatial location in the tissue. Given the exciting developments in the field of mass cytometry, this technology is primed to be a powerful tool to investigate the RTK signalling heterogeneity in tumour specimens as well as provide single-cell resolution to RTK signalling analysis in cell-culture experiments.

## 5. Concluding remarks   

Over the past decade, advances in sample preparation, phosphopeptide enrichment, data-acquisition methods and new MS instrumentation have increased our knowledge of the mechanisms of RTK regulation and signalling. Despite this progress, there are a significant number of areas in RTK biology which have yet to be fully explored. The function and signalling of a large number of RTK family members is still poorly characterized. There is much to be learned about the temporal signalling kinetics of many of these receptors and the degree of co-activation and crosstalk between individual RTKs. There is also a need to investigate RTK spatial distribution, both in terms of heterogeneous receptor expression and activation in tissue, and intracellular RTK compartmentalization. State-of-the-art MS approaches such as those discussed in this review directly address some of the current gaps in our knowledge. Coupled with effective computational modelling and integration of disparate data sources, including proteomic, transcriptomic and epigenomic information, with techniques such as the prize-collecting Steiner forest and tree approach (Huang *et al.*, 2013 ▸; Tuncbag *et al.*, 2013 ▸), these new strategies will be critical in ushering in a new era of RTK research. The use of these technologies over the next decade will undoubtedly yield exciting new insights into RTK signalling and reveal the impact of deregulating these critical networks in diseases such as cancer.
